# Editorial: Cardiac Pacemaking in Health and Disease: From Genes to Function

**DOI:** 10.3389/fphys.2022.913506

**Published:** 2022-05-30

**Authors:** Alicia D’Souza, Gerard J. J. Boink, Futoshi Toyoda, Pietro Mesirca

**Affiliations:** ^1^ Division of Cardiovascular Sciences, University of Manchester, Manchester, United Kingdom; ^2^ Departments of Cardiology and Medical Biology, Amsterdam University Medical Centers, Location University of Amsterdam, Amsterdam, Netherlands; ^3^ Amsterdam Cardiovascular Sciences, Research Program: Heart Failure and Arrhythmias, Amsterdam, Netherlands; ^4^ Department of Physiology, Shiga University of Medical Science, Otsu, Japan; ^5^ Institut de Génomique Fonctionnelle, Université de Montpellier, CNRS, INSERM, Montpellier, France; ^6^ LabEx Ion Channels Science and Therapeutics, Montpellier, France

**Keywords:** pacemaker tissue, ion channels, atrial arrhythmia, channelopathies, heart

The origin of the heartbeat and the intricacies of cardiac pacemaker automaticity have captivated the interest of physiologists for over 70 years. This continues to be a vibrant and innovative area of research, where state-of-the-art imaging, genomic, transgenic and bioengineering modalities, coupled with classical electrophysiological and computational techniques have enabled unprecedented insight into the fundamentals of automaticity. This Research Topic is intended to hold up a mirror to the current activities in the field; we invited commentaries and original contributions from leading experts on themes relating to the functional or developmental aspects of pacemaker activity and its dysregulation in the context of conduction system disease.

In this Research Topic, DiFrancesco evocatively considers landmark discoveries that underscored the concept of “funny” (f-) current (*I*_f_)-based pacemaking and summarises the current and future clinical applications of these findings. These include the development of Ivabradine (Procolaran^©^), an *I*_f_ blocker widely prescribed as a heart rate lowering therapy for stable angina and heart failure, and the potential for biological pacemaking based on transfer of HCN channels (the molecular basis of the *I*_f_ current) in the diseased conduction system. An important characteristic of *I*_f_ is its direct regulation by cyclic adenosine monophosphate (cAMP), and in the second article, Porro et al. review our current understanding of the structure-function relationships that underlie cAMP-mediated changes in HCN conformation and gating. The authors contextualise their exciting recent advances in deciphering how binding of cAMP to the Cyclic Nucleotide-Binding Domain changes HCN-channel conformation via interaction with cytoplasmic C-terminal C-linker. Moreover they highlight how detailed insight into channel structure has led to the development of novel peptide tools such as TRIP8b_nano_ (Saponaro et al., 2018) enabling selective inhibition of cAMP dependent HCN regulation, sparing inhibition of channels’ conductance compared to traditional pore blockers. In the same field, Hennis et al. provide a contemporary review on heart rate modulation in the sinus node. The authors cite a large body of evidence contrasting the traditional view that cAMP-dependent HCN4 regulation mediates chronotropic responses, and instead postulate upon a role for cAMP-mediated PKA-dependent phosphorylation of Ca^2+^ clock proteins integral to pacemaker activity in this process. Based on their recent elegant work (Fenske et al., 2020) the authors propose that the role of HCN4 is not to orchestrate the heart rate response to autonomic input, as commonly thought, but rather to determine the intrinsic HR and stabilise it. In this work, firing and non-firing/dormant myocytes were detected in the mouse sinus node and it was determined that cAMP-dependent HCN4 activation regulates a “tonic entrainment” process between firing and dormant cells that can slow down the predominant rhythm. The concept that dormant sinus node myocytes have a physiological relevance is an intriguing one that has received attention in recent years (Kim et al., 2018; Tsutsui et al., 2018; Bychkov et al., 2020) and in this Research Topic the Lakatta group in Tsutsui et al. delve further into their study. The team confirm and expand their previous observation that adrenergic stimulation revives spontaneous activity in dormant sinus node myocytes, to maximum firing rate similar to that of spontaneously firing myocytes. Mechanistically, this work demonstrates an underlying role for cAMP-mediated augmentation of *I*_CaL_, *I*_K_ and *I*_f_ densities, an increase in diastolic local calcium release, alongside phosphorylation of the critical SR “Ca^2+^-clock” protein phospholamban for obtaining high rates of firing in previously dormant myocytes. These data offer new insight into how the sinus node reaches high pacemaking frequencies by synchronising dormant and firing cell activity.

Outside cAMP-dependent regulation, new signalling pathways that modulate heart rate independently from the autonomic nervous system have been uncovered in recent years. In this regard, new roles for protein kinase-dependent pathways in the regulation of HCN channel availability are described in a research paper by Ira Cohens’ team (Lu et al.). Building upon their previous work determining a role for phosphatidylinositol-3-phosphate kinase (PI3K) in sinus node rate by modulation of basal *I*_*f*_ half-activation (Lin et al., 2019), the team demonstrate PI3K-dependent AKT phosphorylation of HCN2 channels at the Serine 861 residue positively regulates HCN2-mediated *I*_*f*_.

Another important factor potentially regulating pacemaker activity on a beat-to-beat basis–independently of autonomic input–is mechanical force. Cyclical stretching of atrial tissue during the cardiac cycle is predicted to regulate stretch-activated channels in several cell types in the sinus node, including pacemaker cells, fibroblasts and endothelium (MacDonald et al., 2020; Quinn and Kohl, 2012, 2021). The article by Turner et al. reports, for the first time, the expression profile of mechanosensitive ion channels in murine sinus node indicating potential effectors of fast acting mechanical forces: Piezo-1, Trek1 and Ca^2+^ activated K^+^ (BK) channels that show significant expression in the sinus node in comparison to its molecular marker Hcn4. In addition, the article suggests new potential candidates for stretch-dependent regulation of pacemaker activity and discusses the possible mechanisms of interplay between mechanosensitive channels and the current model of sinoatrial automaticity in relation to the proposal of the “mechanical clock” by Quinn and Kohl (2021).

The functional role of K^+^ channels in pacemaker activity has been hotly debated. Indeed, K^+^ channels critically contribute to the balance between inward and outward currents during the automatic action potential cycle. Small-conductance calcium activated K^+^ (SK1-3) channels play an important role in automaticity by matching necessary outward current with intracellular calcium dynamics. However, excessive calcium release can induce inhibition of pacemaking by over-activation of SK channels. Next, a review article by Weisbrod discusses expression and pathophysiological roles of SK channes in the heart - an important summary of the known functional properties of these channels, also highlighting their therapeutic potential in cardiac arrhythmias and in heart failure.

Numerical modelling of pacemaker activity has proved to be an important tool to widen mechanistic understanding of automaticity by *in silico* manipulation of ion channels and proteins controlling intracellular calcium dynamics. Five computational articles in this Research Topic provide new insights into pacemaker activity. First, the study by Yang et al. addresses an important question arising from the existence of electrical and calcium dependent oscillators in pacemaker cells. This manuscript suggests that the degree of self-organisation of RyR-dependent calcium release sites underlies variability in the pacemaker interbeat, by generating oscillations of the Na^+^/Ca^2+^ exchanger current (I_NCX_). The activity of NCX thus reflects the degree of organisation of the calcium oscillator. Since NCX also couples to membrane voltage, this phenomenon explains the intrinsic variability of sinus node cell firing under both basal conditions and autonomic activation.

Next, two articles by the Zhang group combine mathematical models with previously reported experimental data to provide mechanistic insight into the physiological modulation of sinus node function across the lifespan. Presenting an updated computational model for neonatal rabbit sinus node myocytes, Alghamdi et al. investigate how ion channel remodelling may explain differences in pacemaking between neonatal and adult sinus node myocytes and their response to acetylcholine. In follow on studies the authors utilize a mathematical model of the rat sinus node action potential to interrogate the mechanisms by which sinus node function slows in advanced age, arriving at a predominant role for *I*_*Ca*_,_*L*_, remodelling in age-related sinus bradycardia (Alghamdi et al.). Zhang and colleagues also demonstrate the utility of multiscale modelling in improving understanding of atrial fibrillation susceptibility following a gain-of-function mutation in the transcription factor Pitx2c (Bai et al.) and in an additional article (Bai et al.) the authors apply numerical simulations and theoretical analysis to provide new insight into the mode of action of *I*_f_ block with clinical concentrations of ivabradine.

The range and scope of approaches in the aforementioned articles demonstrate the advanced resolution and level of detail at which the electrophysiological basis of pacemaker automaticity is currently interpreted. Less well-established are the precise transcriptional and epigenetic mechanisms that underpin pacemaker cell development and function–an important knowledge gap given that genetic variation in regulatory elements modifies developmental and disease gene expression patterns and phenotypes. In this Research Topic Mandla et al. explore how recent progress in chromatin biology, bioinformatics, and human genetics has enabled unique insight into transcriptional regulators and genomic loci critical for pacemaker cell development. The authors discuss recent exceptional work defining the sinus node cis-regulatory landscape (Fernandez-Perez et al., 2019; Van Eif et al., 2019; Galang et al., 2020) and the validation of novel enhancers, such as the pacemaker cell-specific enhancer for Islet1 (Galang et al., 2020), essential for development and function of the sinus node. Further dissection and insight into such regulatory pathways may have important implications in stratifying patients at risk for rhythm disturbances and pacemaker implant, aid understanding of sinus node dysfunction (SND) pathophysiology and identify novel approaches for sinus node regeneration and therapy.

SND mechanisms and new therapies for its rescue are of considerable interest in the field, and the focus of three original research articles in this Research Topic. Végh et al. explored stem cell based delivery of the well-established biological pacemaker transgene combination HCN2/SkM1 (Boink et al., 2013). Here, HCN2 is overexpressed to augment diastolic depolarization rate, while the skeletal muscle sodium channel, SkM1, can hyperpolarize action potential threshold, thereby accelerating and stabilizing the induced biological pacemaker rhythm. In the current study the authors used cardiac myocyte progenitor cells (CMPCs) as a new gene delivery platform to potentially induce long-term biological pacing (Végh et al., 2019). In doing so they optimized non-viral, electroporation-based manipulation of CMPCs, which appeared to be superior over *ex vivo* lentiviral transduction. Moreover, their computational simulation studies identified an SkM1-mediated increase in final stage diastolic depolarization as a novel mechanism-of-action for biological pacing based on HCN2/SkM1. Soattin et al. investigated subsidiary atrial pacemakers in a large animal (goat) model of SND. Radiofrequency ablation of the sinus node resulted in pacemaking from subsidiary sites in a proportion of animals, leading the authors to speculate upon its role as a dominant pacemaker in SND. Finally, Bidaud et al. demonstrate that genetic ablation of G-protein-gated inwardly rectifying potassium channels (responsible for *I*_KAch_ current) counteracts slowed pacemaking induced by exercise training in mice by preventing transcriptional and electrical remodelling of pacemaking ion channels. This work builds on findings by D’Souza and Boyett demonstrating that a microRNA network orchestrates reduced sinus node ion channel expression in a range of situations where dysfunctional pacemaking is observed, including exercise training (D'Souza et al., 2014; D'Souza et al., 2017; Mesirca et al., 2021) and heart failure (Yanni et al., 2020). Based on the observations ranging from the whole animal to the microRNA, coupled with previous work by the team (Mesirca et al., 2014; Mesirca et al., 2016) it is concluded that *I*_KAch_ modulation may be an effective pharmacological strategy for SND rescue.

In sum, the articles in this Research Topic offer a wealth of insight into basic sinus node automaticity while highlighting new themes in its development, physiology and adaptation to disease. The challenge for the field (as for writing concluding remarks!), is how to distil diverging and ever-evolving appreciation of fundamental sinus node biology into the major outstanding questions that preclude identification of new therapeutic targets for SND–a disease that will become increasingly more common as the population ages, and for which palliation by pacemaker implant is the only available therapeutic option. The articles in this Research Topic reiterate that normal pacemaking arises from interactions between the ion channel/Ca^2+-^ handling ensemble, signal transduction pathways (e.g., PI3K) and genetic/epigenetic influences. Further adding to the complexity are interactions between dormant and firing myocytes and increasingly well-recognized myocyte-nonmyocyte (e.g., fibroblast and macrophage) crosstalk. In the era of precision medicine, our ability to integrate and follow these networks *in vivo* in refined SND models with multiomics technologies and multiscale computer modelling will become as important as our capacity to dissect the role of each component. In the meantime, gene therapy (with HCN2/SkM1 or microRNAs) and pharmacological *I*_KAch_ inhibition show particular promise as emergent therapies for SND management.
